# Libraries

**Published:** 1889-04

**Authors:** 


### EDITORIAL.

### Libraries.

The question often arises in the mind of the most thoughtful and studious of our profession, How many of those practising dentistry keep up with the current literature? How many have any considerable knowledge of what has been written on the leading practical and scientific subjects pertaining to dentistry?

Some estimate may be formed in regard to this, by noting the interest taken in, and the efforts made to secure and use libraries. Doubtless were an investigation to be made, the fact would warrant the statement that a large proportion of the practitioners of dentistry have not even an apology for a library; they even have no care for the journals (that too often go to them without money and without price), after they have scanned the advertising sheets, but permit them soon to be destroyed; some have actually burned large packages of them, as they said to get them out of the way.

And how few are there who have even the ordinary text books, to which they might so often refer with profit. Has this state of things anything to do with the fact that not more than one in ten of those engaged in the practise of dentistry ever engage in association work, or even attend associations, or do anything for the development or building up of that profession, on the ragged edge of which they have been hanginge-xcrescences—living on it, yet contributing nothing to it.

And further, Is here found an explanation to the fact, that of those who do attend associations not more than one in five take any part, or add an iota to the interest except to play the sponge, and to stand up and be counted?

It is better that such should be present than to be absent; but it would be better still if they would prepare themselves to add something to the interest of the association, and for the benefit of their brethren.

Now as to the book question: It may safely be said that not more than one in five hundred of the dentists of this country have any interest, or desire to possess a good dental library. Fortunately the attention of a few are being devoted to this subject with commendable energy ; and as a result perhaps from six to ten libraries, with a good degree of completeness have been collected in this country ; but owing to long delay, this work has been attended with very great difficulty, and many things that in bygone years were easily attainable, can not now be procured at all. Irreparable loss has thus been sustained by the profession through want of interest and appreciation. One of the first objects of an educational institutional should be the establishment of a good library ; but is it not true that not more than two or three dental colleges of this country have even the pretense of a library ? And even those that have were so late in beginning their collection that they fail to include many very desirable books, because they are out of print and are not obtainable.

The private libraries of dental books that are of any considerable extent are few indeed. Perhaps none have accomplished as much in this direction as Dr. H. J. McKellops, of St. Louis. He has now over 1480 volumes in his collection, and as the number he has not obtained decreases, his energy and determination to get them increases, so that now when he meets a friend the first request is, let me see your books, and if he finds any not in his collection—look out!

Dr. A. L. Northrop, of New York, has also a splendid collection, perhaps well nigh equal to that of McKellep's. Professor Abbott, of New York, it is said, also has a good dental library. Dr. E. G. Betty, of Cincinnati, is also working industriously in that line, but he finds he is going over cleaned ground ; he is trying to make up in energy for the poverty of material. He deserves sympathy in his efforts on ground, that has been worked by McKellops and Northrop.

There is in the Library of the Surgeon General's office at Washington, D. C., quite a large collection of dental works, probably over a thousand volumes. Quite a number of these we have not found in any other library.

While it is well and proper for individuals to make these collections, the public libraries promise more for the future, and indeed serve a much larger purpose for the present than it is possible for a private library to do.

It is certainly important that all material that can be made to serve book collections in the future should be preserved. It is to be hoped that dental colleges will instill into their students a higher appreciation of the literature of the profession than has been entertained in the past.

